# Books and Magazines

**Published:** 1913-03

**Authors:** 


### Books and Magazines.

Golden Rules of Surgery—Volume I of the Golden Rule Series. Especially intended for students, general practitioners, and beginners in surgery.—By Augustus Charles Bernays, A. M., M. D., F. R. C. S., Eng.; Life Member of the German Society for Surgeons of Berlin; Chief Surgeon Lutheran Hospital, and for twenty years Professor of Anatomy and Surgery, St. Louis. Second edition, revised and rewritten by William Thomas Coughlin, M. D., Assistant Professor of Surgery, Chief of Clinic, St. Louis University Medical School, St. Louis. 280 pages. Octavo. C. V. Mosby Co., St. Louis. Price, $2.25.

The entire absorption of a large first edition of the Golden Rules of Surgery made necessary the issue of the present one. Its enlargement and elaboration by the junior author has made it possible to cover the entire field of surgery in a thorough and systematic manner, at the same time preserving the character and charming style that made the first edition of this book popular.

A Reference Handbook of the Medical Sciences, embracing the entire range of Scientific and Practical Medicine and Allied Science. By various writers. Third edition, completely revised and rewritten. Edited by Thomas Lathrop Stedman, A. M., M. D. Complete in eight volumes. Volume I. Illustrated by numerous chromolithographs and six hundred and eleven fine half-tone and wood engravings. Nine hundred and thirty-six pages. Imperial quarto. The set: muslin, $56; leather, $64; half morocco. $72 (subscription). Wm. Wood & Co., Publishers, New York.

The "Reference Handbook" is too well known by the medical profession of this country to require very much introduction. The first two editions were edited by Dr. Albert H. Buck, and their sale unquestionably exceeded that of any other medical work of like importance ever published. Written by over five hundred men of acknowledged eminence in their various branches, and arranged in form midway between that of the encyclopedia and the dictionary, this book has proved exceedingly useful to its possessors and extraordinarily popular with the medical profession.

The third edition is practically a new work, for not only have the articles dealing with subjects treated in the second edition been thoroughly revised, and in many cases rewritten, but there are also more or less extended treatises on the very large number of new subjects. Numerous short descriptive articles on subjects not calling for encyclopedic treatment, yet demanding more than a mere definition, have been included, and a series of brief biographical sketches, giving the salient points in relation to the lives and works of the leaders of medical thought in all parts of the world's history. The editor has endeavored in every way to enhance the value of the "Reference Handbook" as a work of convenient and dependable reference for practitioners and students of medicine.

Volume I contains within its nine hundred and thirty-six pages all the titles from "Aachen" to "Bacteriuria." It is very well printed, on paper which shows the cuts well, but has not that shiny surface so trying to the eyes, especially when reading by artificial light.

Dr. Stedman, the editor of the new edition of the "Reference Handbook," is well known as the editor of the *Medical Record,* and of *Twentieth Century Practice,* and as the author of Stedman's Medical Dictionary.

